# Head-to-head comparison of qSOFA and SIRS criteria in predicting the mortality of infected patients in the emergency department: a meta-analysis

**DOI:** 10.1186/s13049-018-0527-9

**Published:** 2018-07-11

**Authors:** Jianjun Jiang, Jin Yang, Jing Mei, Yongmei Jin, Youjin Lu

**Affiliations:** Department of Respiratory Medicine, the Second Affiliated Hospital of Anhui Medical University, Hefei, China

**Keywords:** SIRS, qSOFA, Infection, Emergency department, Mortality, Prognosis

## Abstract

**Background:**

Recently, the concept of sepsis was redefined by an international task force. This international task force of experts recommended using the quick Sequential Organ Failure Assessment (qSOFA) criteria instead of the systemic inflammatory response syndrome (SIRS) criteria to classify patients at high risk for death. However, the added value of these new criteria in the emergency department (ED) remains unclear. Thus, we performed this meta-analysis to determine the diagnostic accuracy of the qSOFA criteria in predicting mortality in ED patients with infections and compared the performance with that of the SIRS criteria.

**Methods:**

PubMed, EMBASE and Google Scholar (up to April 2018) were searched for related articles. A 2 × 2 contingency table was constructed according to mortality and qSOFA score (< 2 and ≥ 2) or SIRS score (< 2 and ≥ 2) in ED patients with infections. Two investigators independently assessed study eligibility and extracted data. We used a bivariate meta-analysis model to determine the prognostic value of qSOFA and SIRS in predicting mortality. We used the I^2^ index to test heterogeneity. The bivariate random-effects regression model was used to pool the individual sensitivity, specificity, diagnostic odds ratio (DOR), positive likelihood ratio (PLR), and negative likelihood ratio (NLR). The summary receiver operating characteristic curve (SROC) was constructed to assess the overall diagnostic accuracy.

**Results:**

Eight studies with a total of 52,849 patients were included. A qSOFA score ≥ 2 was associated with a higher risk of mortality in ED patients with infections, with a pooled risk ratio (RR) of 4.55 (95% CI, 3.38–6.14) using a random-effects model (I^2^ = 91.1%). A SIRS score ≥ 2 was a prognostic marker of mortality in ED patients with infections, with a pooled RR of 2.75 (95% CI, 1.96–3.86) using a random-effects model (I^2^ = 89%). When comparing the performance of qSOFA and SIRS in predicting mortality, a qSOFA score ≥ 2 was more specific; however a SIRS score ≥ 2 was more sensitive. The initial qSOFA values were of limited prognostic value in ED patients with infections.

**Conclusions:**

A qSOFA score ≥ 2 and SIRS score ≥ 2 are strongly associated with mortality in ED patients with infections. However, it is also clear that qSOFA and SIRS have limitations as risk stratification tools for ED patients with infections.

**Electronic supplementary material:**

The online version of this article (10.1186/s13049-018-0527-9) contains supplementary material, which is available to authorized users.

## Background

Sepsis is a common cause of critical illness and mortality worldwide [1, 2], accounting for 10% of intensive care unit (ICU) cases, and it has an in-hospital mortality rate of 10 to 20% [3–5]. However, both the risk stratification in patients with acute infections and the identification of sepsis are still challenges for clinicians. Therefore, a reliable method for evaluating the severity of sepsis may help clinicians determine whether aggressive therapy and close monitoring are more appropriate than conservative therapy, thus improving patients’ initial management and, ultimately, survival.

The previous consensus definitions of sepsis required an infection and two or more systemic inflammatory response syndrome (SIRS) criteria [6, 7]. Four SIRS criteria were defined, namely, tachypnea (respiratory rate > 20 breaths per minute), tachycardia (heart rate > 90 beats per minute), leukopenia or leukocytosis (leucocyte count > 12,000 cells/μL or < 4000/μL), and fever or hypothermia (body temperature > 38 °C or < 36 °C, respectively). Recently, this syndrome was redefined by an international task force in the third international consensus definitions for sepsis and septic shock (sepsis-3) [8]. Furthermore, the new definition has discarded the concept of SIRS. A new set of criteria, included in the quick Sequential Organ Failure Assessment (qSOFA), was introduced in sepsis-3 (range, 0–3; each receiving 1 point if the following criteria are met: systolic arterial blood pressure ≤ 100 mmHg; respiratory rate > 21 breaths/min; or altered mental status). A qSOFA score of 2 or higher was associated with a higher risk of mortality. The definition group stated that qSOFA was a better predictor of mortality than SIRS and recommended using a qSOFA score of ≥2, instead of a SIRS score of ≥2, to identify infected patients at high risk for death. However, the added value of these new criteria in specific clinical settings remains unclear, including in the emergency department (ED).

We included all studies that compared qSOFA and SIRS scores in ED patients with infections and performed a meta-analysis of the available studies to determine the diagnostic accuracy of the qSOFA criteria in predicting mortality in ED patients with infections. The performance of the qSOFA criteria was compared with that of the SIRS criteria.

## Methods

This meta-analysis was performed in accordance with the Preferred Reporting Items for Systematic Reviews and Meta-Analyses (PRISMA) statement.

### Search strategy and selection criteria

We systematically searched the literature using PubMed, EMBASE and Google Scholar before April 2018. The search strategy was as follows: (“quick Sequential Organ Failure Assessment” or “qSOFA”) and (“Systemic Inflammatory Response Syndrome” or “SIRS”) and (“infection” or “infections”) and (“Emergency Department” or “ED”). The search was restricted to studies written in English. To ensure a comprehensive literature search, we also examined the references of the included articles.

Two investigators (Jianjun Jiang and Jin Yang) independently screened and included eligible studies, and any disagreement was resolved by group consensus. The inclusion criteria were the following: (1) the study population included ED patients with infections, (2) a clear diagnostic reference standard for infection was used, (3) the purpose was to evaluate or compare the prognostic value of qSOFA and SIRS in predicting death within the same patient population, and (4) adequate information had to be provided to build a 2 × 2 contingency table (true positives [TP], false positives [FP], false negatives [FN], and true negatives [TN]). The exclusion criteria were as follows: review articles, letters, conference abstracts, and expert opinions.

### Data extraction and quality assessment

The following data were extracted from the original studies: first author, year of publication, study design, country of origin, sample size, prevalence of mortality, endpoint, mean ages, ratio of male patients, time point of score calculation, TP, FP, FN, and TN. We contacted authors with any items that required clarification. To assess quality, modified criteria based on the criteria of Hayden et al. were used [9](Additional File 1). We assessed the following six items: (1) population, (2) follow-up, (3) measurement of severity scores, (4) outcome measurement, (5) confounding variables, and (6) statistical analysis. Each item was scored from 0 to 2, and the total scores ranged from 0 to 12. When publications had scores ≥9, the methodological study design was considered acceptable.

### Statistical analysis

Statistical analysis was conducted using the MIDAS module of the STATA software, version 12.0 (Stata Corporation, College Station, TX), Meta-Disc 1.4 (XI Cochrane Colloquium, Barcelona, Spain) and RevMan5.3 (Nordic Cochrane Center, Copenhagen, Denmark). We identified TP, FN, FP, and TN based on the effects of qSOFA (scores < 2 and ≥ 2) or SIRS (scores < 2 and ≥ 2) on all-cause mortality. Relative risk (RR) was used to evaluate the predictive value of qSOFA and SIRS, which was pooled by random-effects or fixed-effects models, according to DerSimonian and Laird’s method [10]. I^2^ statistics were calculated to assess statistical heterogeneity; I^2^ values of more than 50% indicated a significant level of heterogeneity [10]. If I^2^ was > 50%, the random-effects model was chosen; otherwise, the fixed-effects model was used.

The pooled sensitivity, specificity, diagnostic odds ratio (DOR), positive likelihood ratio (PLR) and negative likelihood ratio (NLR) were calculated using a bivariate random-effects regression model [11, 12]. A summary receiver operating characteristic curve (SROC) was generated to assess the overall diagnostic accuracy [13].

We conducted a subgroup analysis to explore the main source of heterogeneity and explore the prognostic accuracy of qSOFA and SIRS when studies were restricted to different follow-up periods (30-day mortality and in-hospital mortality), used different time points to calculate scores (using the earliest measured clinical values or the worst values to calculate qSOFA and SIRS scores), or were prospective or retrospective studies only. Publication bias was analysed by Deek’s funnel plot.

## Results

**Fig. 1:**
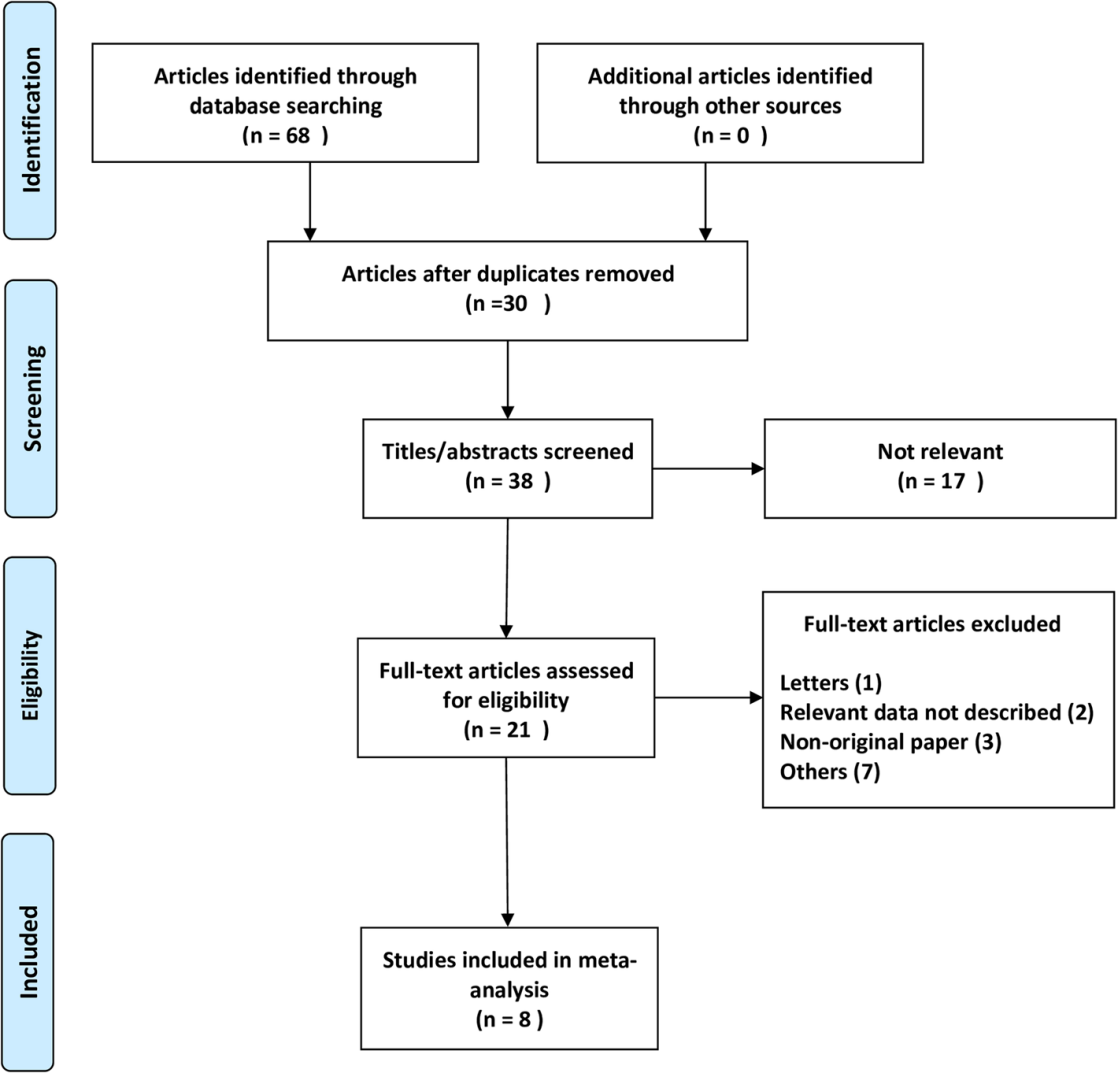
Flow diagram of the study selection process

Our database search retrieved 68 articles. According to the exclusion and inclusion criteria, 8 studies [14–21] met our eligibility criteria and were included in the analysis (Fig. 1). In one study, the authors reported the diagnostic accuracy for two groups of patients separately; therefore, we divided the results of the study into two parts. Thus, we analysed 9 datasets. No additional relevant articles were identified in the bibliographies of the original articles.

### Study characteristics

The main characteristics of the included studies are shown in Table 1. The eight included studies were published between 2017 and 2018. Five studies [14–17, 20] were conducted in Europe, two [18, 21] were conducted in the United States, and one [19] was conducted in Australia. All the studies were carried out in EDs and published in English. The proportion of men varied between 50 and 62%, and the mortality rates varied from 4.4 to 14.6% in these studies. The most frequent site of infection was the respiratory system. The majority of the studies used in-hospital mortality or 30-day mortality as their primary outcome measure. Five cohorts [16, 17, 20, 21] were prospective, observational cohort studies, and four [14, 15, 18, 19] studies were described as retrospective. Among these datasets, five [14–18] used the first set of available laboratory values and vital signs to calculate the SIRS and qSOFA scores, and four [19–21] studies used the worst values during the ED stay to calculate the SIRS and qSOFA scores.

**Table 1:** Study characteristics

Author/year	Study design	Country	Sample size (n)	Age (mean)	Male (%)	Prevalence of mortality (%)	Time of score calculation	Measured mortality	Participant selection
Goulden/2018	Retrospective	UK	1818	68	51	14.6	ED arrival	In-hospital mortality	Suspected infection
Ranzani/2017	Retrospective	Spain	6874	66	62	6.4	ED arrival	In-hospital mortality	Community-acquired pneumonia
González Del Castillo/2017	Prospective	Spain	1071	84	51	6.5	ED arrival	30-day mortality	Suspected or confirmed infection
Askim/2017	Prospective	Norway	1535	62	53	4.4	ED arrival	30-day mortality	Suspected infection
Moskowitz/2017	Retrospective	USA	24,164	64	51	4.9	ED arrival	In-hospital mortality	Suspected infection
Willams/2017	Retrospective	Australia	8871	49	51	8.7	Worst values during ED stay	30-day mortality	Suspected infection
Freund/2017	Prospective	Europe	879	67	53	8.4	Worst values during ED stay	In-hospital mortality	Suspected infection
Henning [a]/2017	Prospective	USA	4618	57	52	4.2	Worst values during ED stay	In-hospital mortality	Suspected infection
Henning [b]/2017	Prospective	USA	2132	57	52	3.9	Worst values during ED stay	In-hospital mortality	Suspected infection

### Quality assessment and publication bias

**Fig. 2:**
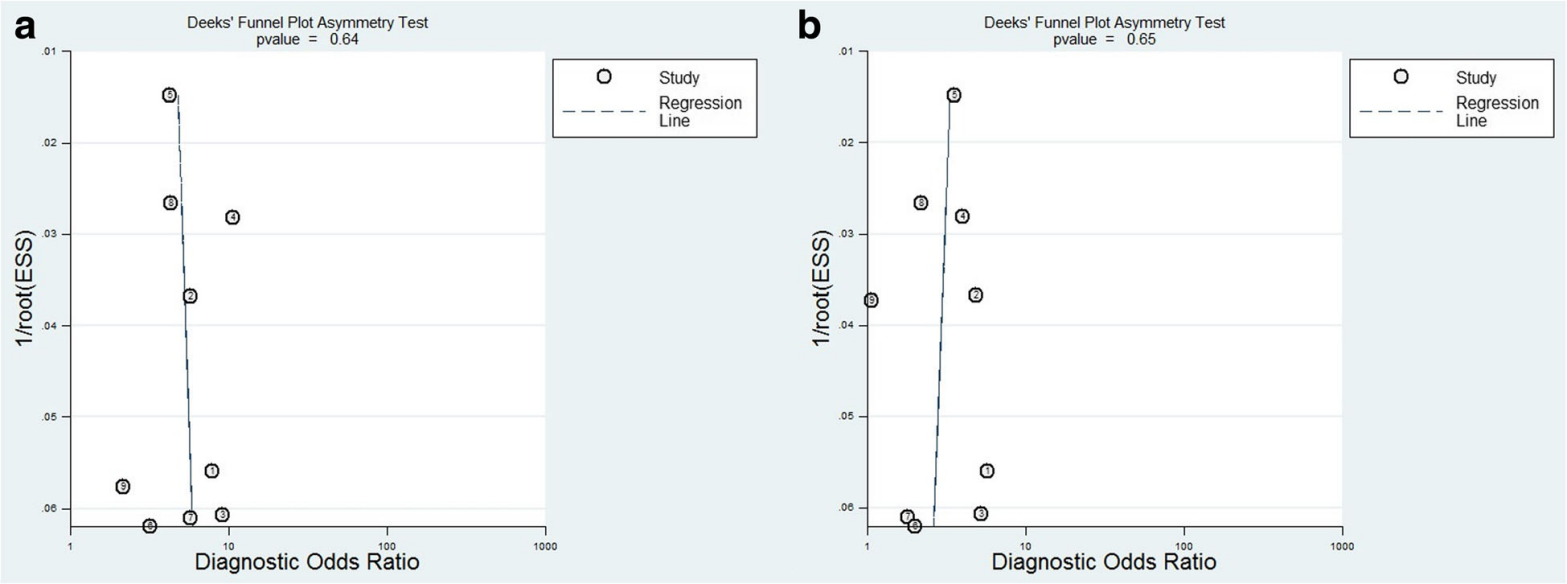
Deek’s funnel plot of publication bias (**a)**. For qSOFA; **b**. For SIRS). Potential publication bias exists (*P* < 0.05)

We conducted a quality assessment based on the criteria developed by Hayden et al. [9]. Six studies [14, 15, 18–21] were considered to be of moderate quality (9 to 10), and two studies [16, 17] were of good quality (≥11) (Table 2). Deek’s Funnel plot indicated that no publication bias existed (Fig. 2).

**Table 2:** Study quality assessment

Study	Population	Follow-up	Measurement of severity scores	Outcome measurement	confounding variables	Statistical analysis	Quality score(total)
Goulden/2018	2	1	2	2	2	1	10
Ranzani/2017	2	1	1	2	2	2	10
González DelCastillo/2017	2	2	2	2	1	2	11
Askim/2017	2	2	2	2	1	2	11
Moskowitz/2017	2	1	2	2	2	1	10
Willams/2017	2	1	1	2	2	1	9
Freund/2017	2	2	1	1	2	2	10
Henning/2017	2	1	1	2	2	1	9

### Data synthesis and meta-analysis

#### Analysis of the association between qSOFA scores and mortality

**Fig. 3:**
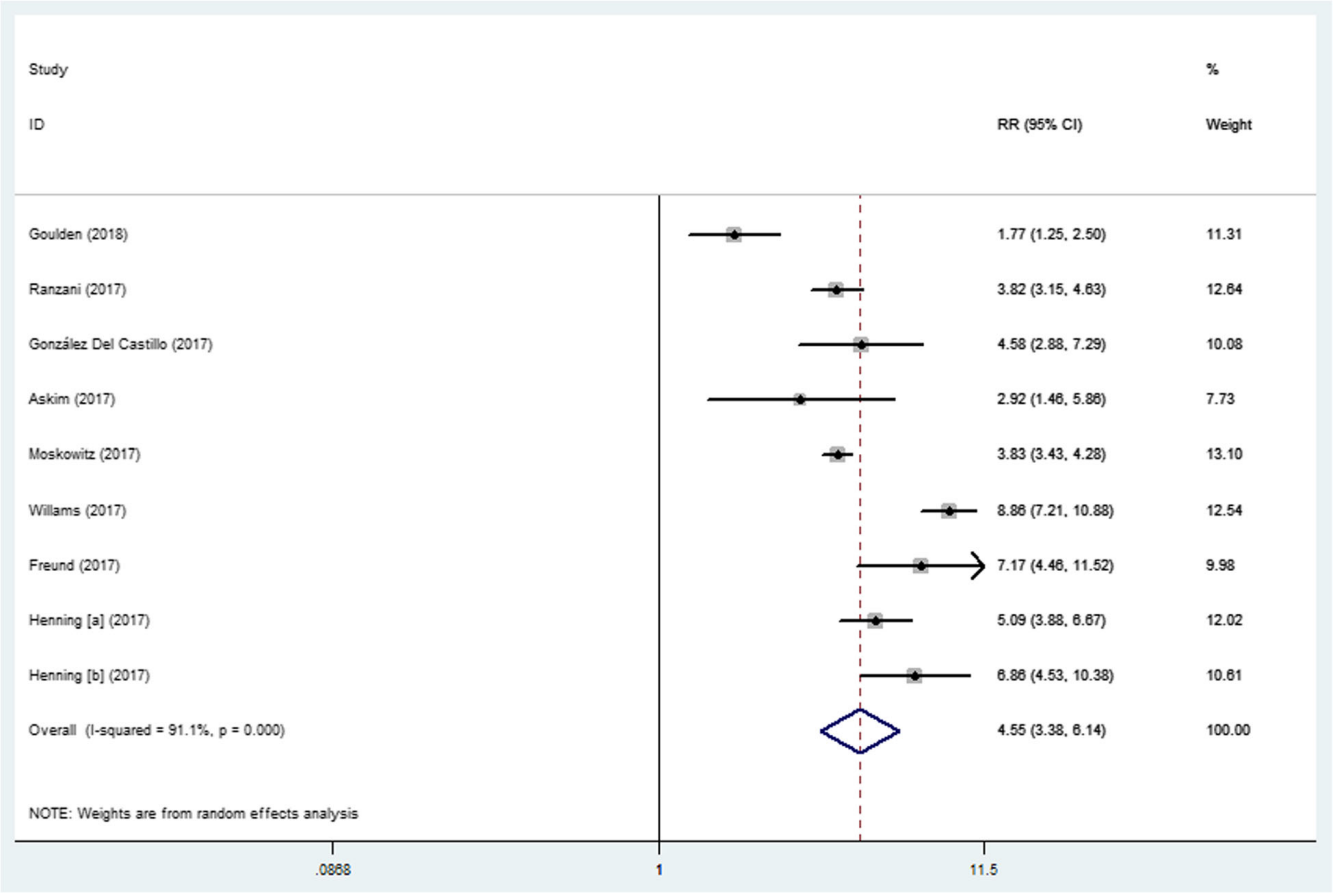
Forest plot of qSOFA scores ≥2 for predicting mortality in ED patients with infections

**Fig. 4:**
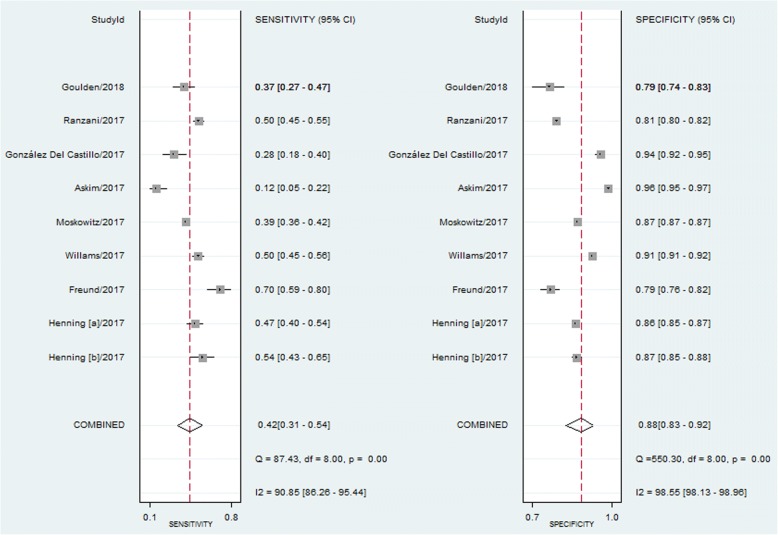
Forest plot of the sensitivity and specificity of qSOFA scores ≥2 for predicting mortality in ED patients with infections

All the included studies indicated that a qSOFA score of ≥2 was associated with a higher risk of mortality in ED patients with infections, with a RR ranging from 1.77 to 8.86. Due to the significant heterogeneity between studies (I^2^ = 91.1%), we used a random-effects model for pooled RR estimates. The pooled RR was 4.55 (95% CI, 3.38–6.14) (Fig. 3). The pooled sensitivity and specificity were 0.42 (95% CI, 0.31–0.54) and 0.88 (95% CI, 0.83–0.92), respectively (Fig. 4). Furthermore, the pooled DOR, PLR, NLR, and AUC were 5 (95% CI, 4–7), 3.5 (95% CI, 2.80–4.40), 0.66 (95% CI, 0.56–0.78), and 0.78 (95% CI, 0.74–0.81), respectively.

#### Analysis of the association between SIRS scores and mortality

**Fig. 5:**
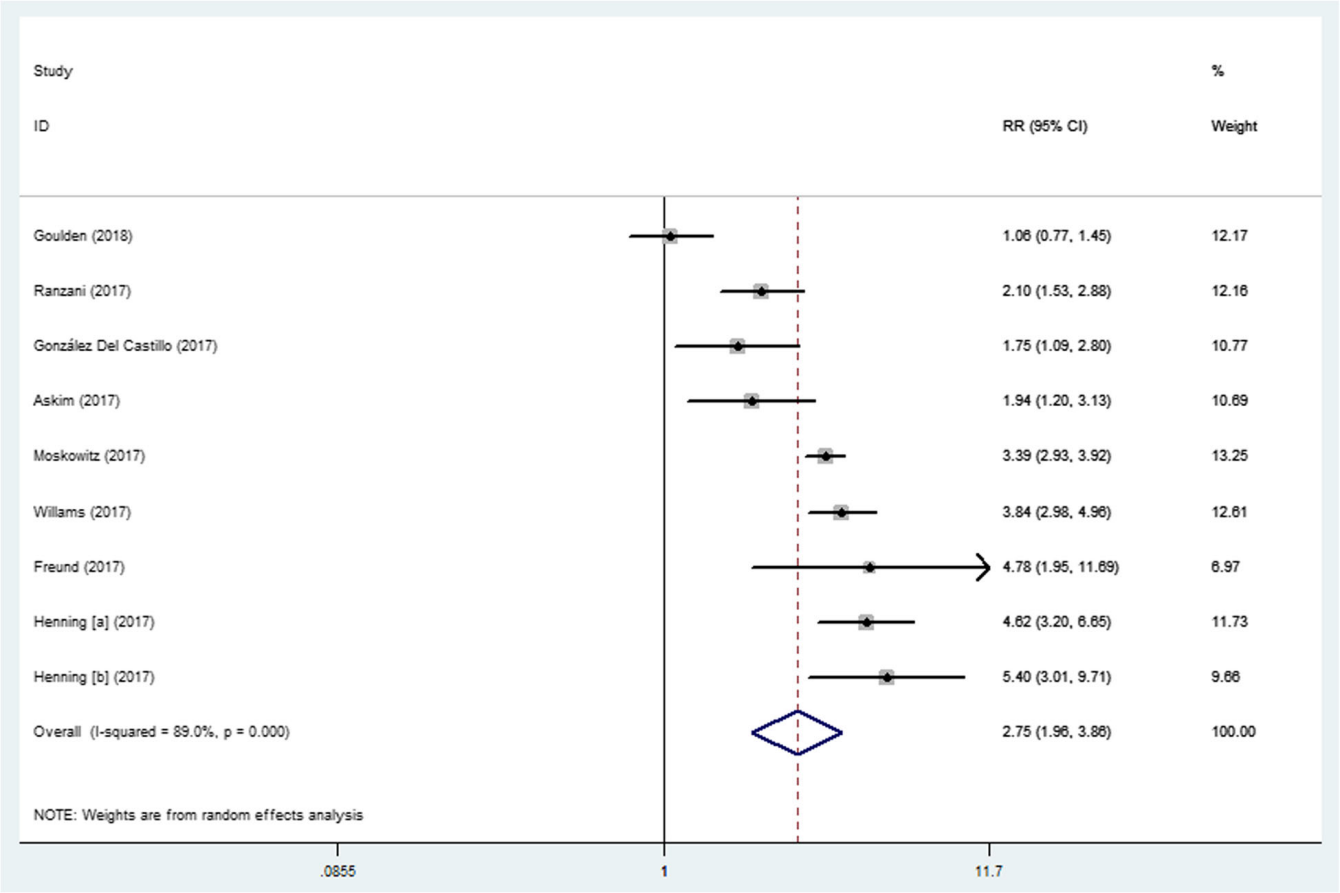
Forest plot of SIRS scores ≥2 for predicting mortality in ED patients with infections

**Fig. 6:**
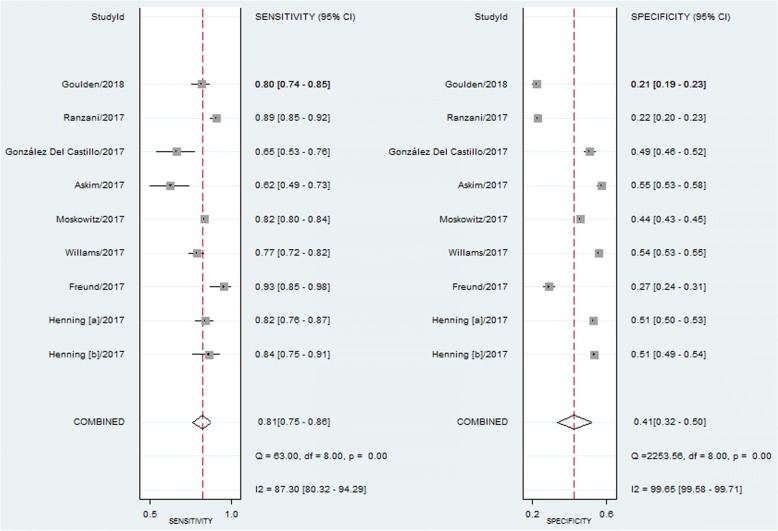
Forest plot of the sensitivity and specificity of SIRS scores ≥2 on predicting mortality in ED patients with infections

Combined data from all eight studies indicated that a SIRS score of ≥2 was associated with a higher risk of mortality, with pooled RR estimates of 2.75 (95% CI, 1.96–3.86) and substantial heterogeneity in the data (I^2^ = 89%) (Fig. 5). The pooled sensitivity and specificity were 0.81 (95% CI, 0.75–0.86) and 0.41 (95% CI, 0.32–0.50), respectively (Fig. 6). Additionally, the pooled DOR, PLR, NLR, and AUC were 3 (95% CI, 2–4), 1.40 (95% CI, 1.20–1.60), 0.47 (95% CI, 0.37–0.59), and 0.70 (95% CI, 0.65–0.73), respectively.

#### Performance comparison of qSOFA and SIRS

**Fig. 7:**
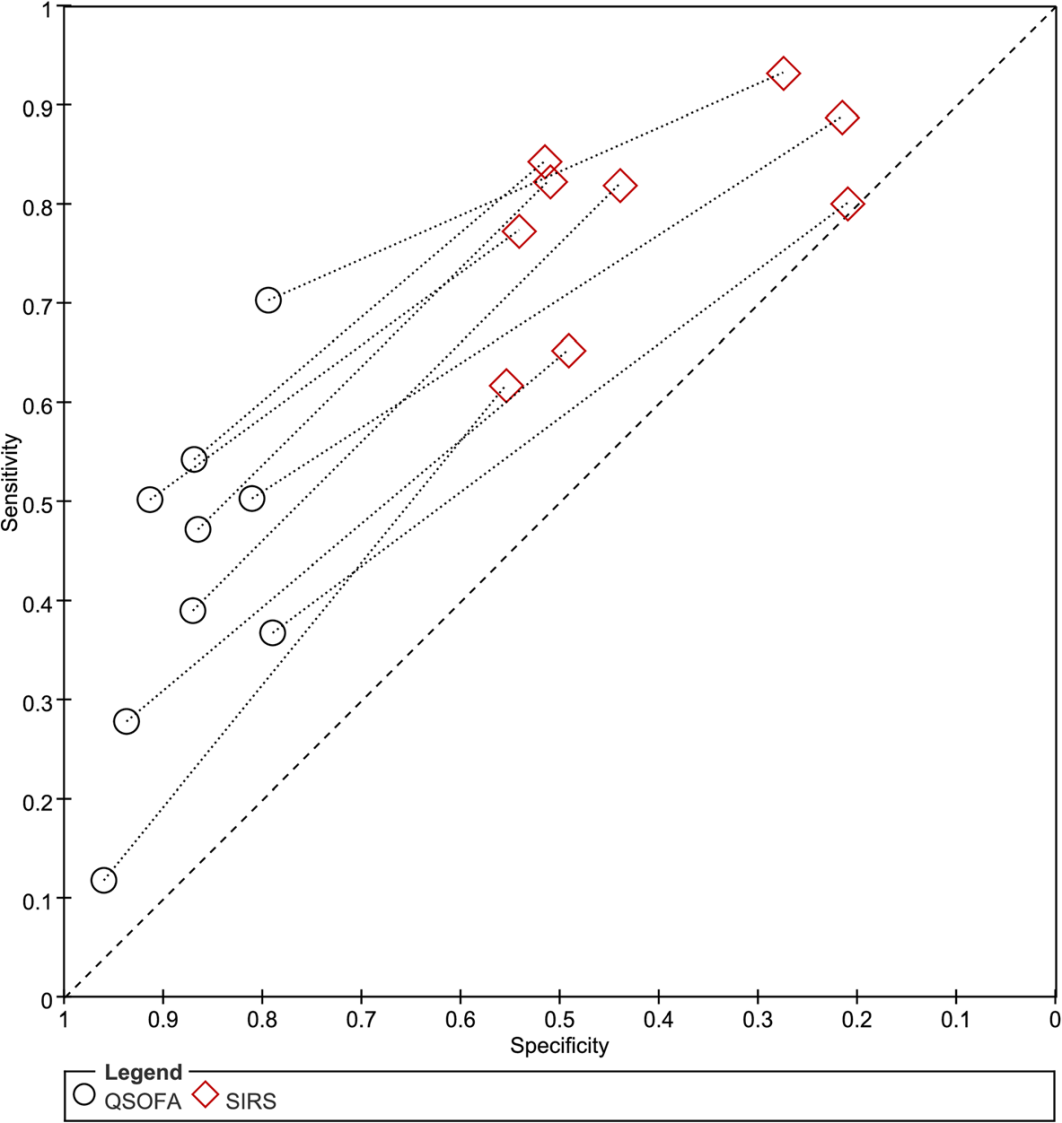
Paired specificity and sensitivity of qSOFA scores ≥2 versus SIRS scores ≥2 in predicting mortality in ED patients with infections

The performance characteristics of qSOFA and SIRS are shown in Table 3. Direct comparisons of data in the studies are shown in Fig. 7. Regardless of whether the comparisons were direct or indirect, they all consistently indicated that a qSOFA score ≥ 2 was more specific, but less sensitive, than a SIRS score ≥ 2 in predicting mortality. As shown in Table 3, qSOFA had a superior positive likelihood ratio compared with that of SIRS, but SIRS had a relatively good negative likelihood ratio.

**Table 3:** Pooled performance characteristics of qSOFA and SIRS criteria for predicting mortality in ED patients with infections

	Sensitivity (95% CI)	Specificity (95% CI)	PLR (95% CI)	NLR (95% CI)	DOR (95% CI)	AUC (95% CI)
qSOFA	0.42 (0.31–0.54)	0.88 (0.83–0.92)	3.5 (2.8–4.4)	0.66 (0.56–0.78)	5 (4–7)	0.78 (0.74–0.81)
SIRS	0.81 (0.75–0.86)	0.41 (0.32–0.50)	1.4 (1.2–1.6)	0.47 (0.37–0.59)	3 (2–4)	0.70 (0.65–0.73)

*qSOFA*, quick Sequential Organ Failure Assessment, *SIRS*, systemic inflammatory response syndrome, *PLR* positive likelihood ratio, *NLR* negative likelihood ratio, *DOR* diagnostic odds ratio, *AUC* area under the curve, *CI* confidence interval

### Subgroup analysis

A subgroup analysis restricted to different follow-up periods, studies using different time points to calculate scores, prospective studies and retrospective studies was performed (Table 4). Studies that used the first measured laboratory values and vital signs to calculate scores had a low sensitivity for qSOFA in predicting mortality in ED patients with infections. Due to the relatively low sensitivity, the initial qSOFA values were of limited prognostic value in ED patients with infections. In addition, studies that used the worst values to calculate qSOFA had a relatively good prognostic performance.

**Table 4:** Subgroup analysis

Subgroup	No. of studies	No. of patients	RR (95% CI)	SEN (95% CI)	SPE (95% CI)	Test for heterogeneity (I^2^)
All studies						
qSOFA	8	52,849	4.55 (3.38–6.14)	0.42 (0.31–0.54)	0.88 (0.83–0.92)	91.1
SIRS	8	52,849	2.75 (1.96–3.86)	0.81 (0.75–0.86)	0.41 (0.32–0.50)	89
Prospective studies						
qSOFA	4	11,122	5.34 (4.19–6.82)	0.39 (0.22–0.60)	0.90 (0.83–0.94)	37.2
SIRS	4	11,122	3.23 (1.95–4.33)	0.80 (0.67–0.89)	0.47 (0.38–0.56)	78.5
Retrospective studies						
qSOFA	4	41,727	3.95 (2.48–6.44)	0.44 (0.38–0.50)	0.85 (0.80–0.90)	86.2
SIRS	4	41,727	2.35 (1.40–3.93)	0.83 (0.78–0.87)	0.34 (0.21–0.49)	84.4
In-hospital mortality						
qSOFA	5	41,372	4.20 (3.18–5.56)	0.49 (0.41–0.57)	0.84 (0.81–0.86)	86.6
SIRS	5	41,372	2.97 (1.84–4.80)	0.85 (0.81–0.88)	0.35 (0.25–0.46)	81.8
30-day mortality						
qSOFA	3	11,477	5.22 (2.70–10.07)	0.41 (0.37–0.46)	0.92 (0.92–0.93)	84.1
SIRS	3	11,477	2.42 (1.40–4.23)	0.74 (0.69–0.78)	0.54 (0.53–0.55)	81.4
The initial values						
qSOFA	5	35,462	3.29 (2.51–4.29)	0.33 (0.22–0.47)	0.90 (0.82–0.94)	79.1
SIRS	5	35,462	1.93 (1.20–3.15)	0.78 (0.68–0.86)	0.37 (0.25–0.51)	92.1
The worst values						
qSOFA	3	17,387	6.89 (5.12–9.27)	0.55 (0.47–0.63)	0.87 (0.82–0.90)	71
SIRS	3	17,387	4.24 (3.50–5.14)	0.85 (0.75–0.91)	0.46 (0.35–0.57)	0

*qSOFA* quick Sequential Organ Failure Assessment, *SIRS* systemic inflammatory response syndrome, *RR* risk ratio, *SEN* sensitivity; *SPE* specificity; *CI* confidence interval

## Discussion

As sepsis is a common cause of critical illness and mortality among infected patients, the early diagnosis and identification of sepsis is paramount to ensure early antibiotic administration and resuscitative therapies. To our knowledge, this is the first meta-analysis and systematic review comparing the performance of the new sepsis definitions with the previous definitions in predicting mortality in ED patients with infections. We first determined that both a qSOFA score of ≥2 and a SIRS score of ≥2 were strongly associated with mortality in ED patients with infections. However, in terms of predicting mortality, qSOFA and SIRS have different trade-offs.

In this meta-analysis, we demonstrated that a qSOFA score of ≥2 was associated with a higher risk of mortality in ED patients with infections. The pooled RR was 4.55 (95% CI, 3.38–6.14), suggesting that a qSOFA score of ≥2 predicted a moderate prognosis for ED patients with infections. We further evaluated the prognostic performance of qSOFA. The high specificity and good positive likelihood ratio of qSOFA are of great value for screening infected patients who are more likely to develop adverse outcomes. Therefore, the qSOFA criteria can be used to urge clinicians to further investigate the presence of organ dysfunction in infected patients, initiate or escalate appropriate therapy, and consider referring patients to the ICU. However, the poorer sensitivity of the qSOFA criteria means that even some patients who are actually at higher risk of death may be incorrectly classified and managed as non-severe. Perhaps the addition of biomarkers, such as lactate, is a possible solution [22, 23]. Notably, when the earliest measured clinical values were used to calculate the qSOFA score, the sensitivity of qSOFA in predicting mortality was quiet low. The initial qSOFA values were of limited prognostic value in ED patients with infections. With regard to the SIRS criteria, we found that a cut-off ≥2 was a prognostic marker of mortality in ED patients with infections, with a pooled RR of 2.75 (95% CI, 1.96–3.86). In terms of predicting mortality, we observed that the SIRS criteria could correctly identify patients who were at a low risk of death and had non-severe infections, with a pooled sensitivity of 0.81 (95% CI, 0.75–0.86) and a pooled NLR of 0.47 (95% CI, 0.37–0.59), thus giving clinicians more confidence in distinguishing patients who may not need to be hospitalized. However, we also observed that the SIRS criteria may lead to more FP, with a pooled specificity of 0.41 (95% CI, 0.32–0.50), thus unnecessarily wasting time and resources.

When comparing the performance of qSOFA and SIRS, qSOFA had a relatively high AUC compared with that of SIRS; however, it should be stressed that the real characteristics of interest for clinical use are the sensitivity and specificity of given cut-off points (e.g., qSOFA score ≥ 2; SIRS≥2). Nevertheless, the AUC can represent the overall discriminatory ability. Regardless of whether comparisons were direct or indirect, they all consistently indicated that a qSOFA score ≥ 2 was more specific but less sensitive in predicting mortality than a SIRS score ≥ 2. Additionally, qSOFA had a superior positive likelihood ratio compared with that of SIRS, but SIRS had a relatively good negative likelihood ratio. Therefore, qSOFA criteria are no better or worse than SIRS criteria in identifying patients with a high risk of death in an ED setting. These risk stratification tools have different strengths and weaknesses. SIRS is a targeted, sensitive screening tool that is well suited for early care and prevention of missed cases. However, the focus of qSOFA is the specific identification of patients at an even higher risk of death, which could aid clinicians in deciding on subsequent care and treatment.

The choice of tool depends on the clinician’s attitude towards resource use and health care, as well as infection-related mortality. In some ED settings, where infection mortality is relatively low and resources are limited, the lower sensitivity of the qSOFA is not a major drawback. Its high specificity and ease of use may help clinicians focus on patients who require more clinical attention. In ED settings that have a relatively high mortality rate of infections and are resource-rich, the most sensitive test (SIRS) may be preferred.

The following limitations of our review should be considered. (1) This meta-analysis revealed significant heterogeneity among the included studies. The studies’ included patients had different infection types, and different outcome measures were used, such as in-hospital mortality or 30-day mortality. Studies used various designs, including prospective and retrospective observational studies, and they used different time points to calculate the scores. Despite these variations, comprehensive subanalyses showed conclusions similar to those of the main analysis. Considering studies that used different time points to calculate scores, different outcome measures, or different designs separately did not influence the conclusions. These analyses have significantly improved homogeneity without affecting the main conclusions. However, despite the multiple subanalyses conducted, the meta-analysis was still influenced by the inherent biases of the included studies and did not display any consistent source to explain the obvious heterogeneity. Some researchers have suggested that there is often a large degree of heterogeneity in meta-analyses of diagnostic studies [24]. We included all articles that directly evaluated qSOFA and SIRS using the same cohort patients to minimize heterogeneity when comparing qSOFA and SIRS. (2) A relatively small number of studies were included, which may not have enabled a complete assessment of the prognostic potential of qSOFA and SIRS.

## Conclusions

In conclusion, our results suggested that both a qSOFA score ≥ 2 and a SIRS score ≥ 2 were strongly associated with mortality in ED patients with infections. However, it is also clear that qSOFA and SIRS have limitations as risk stratification tools for ED patients with infections. qSOFA appears to be a simple, rapid, and effective way to identify patients at high risk for death. However, it seems necessary to identify ways to improve its low sensitivity. Until then, it cannot completely replace the use of SIRS in the ED.

## Additional file

Additional file 1 Hayden’s criteria for quality assessment, modified to apply to studies of infection. (DOCX 18 kb)

## Abbreviations

CI: Confidence interval
DOR: Diagnostic odds ratio
ED: Emergency department
FN: False negatives
FP: False positives
ICU: Intensive care unit
NLR: Negative likelihood ratio
PLR: Positive likelihood ratio
qSOFA: Quick sequential organ failure assessment
RR: Risk ratio
SIRS: Systemic inflammatory response syndrome
SROC: Summary receiver operating characteristic curve
TN: True negatives
TP: True positives
