# Supplementary material for: Head-to-head comparison of qSOFA and SIRS criteria in predicting the mortality of infected patients in the emergency department: a meta-analysis
Source: Scand J Trauma Resusc Emerg Med. 2018 Jul 11;26:56. doi: 10.1186/s13049-018-0527-9 (PMC6042435; doi:10.1186/s13049-018-0527-9)
Supplement: Supplementary file 1 — Hayden’s criteria for quality assessment, modified to apply to studies of infection. (DOCX 18 kb) [file 13049_2018_527_MOESM1_ESM.docx]

Additional file 1: Hayden’s criteria for quality assessment.

| **Hayden’s criteria** | **Components** | **As applied to infection studies** |
| --- | --- | --- |
| 1. Study sample represents the population of interest; design is appropriate to limit potential bias. | 1. Source population clearly defined. 2. Study population described. 3. Study population represents population of interest. | 1. Population limited to infection and excludes other diagnoses. 2. Requires radiological studies,microbiological findings, or clinical context confirmation and uses recognized definitions. 3. Enrols consecutive, unselected patients. 4. Demographics are representative of infection cohorts internationally. |
| 1. Loss to follow-up and study data adequately represent the sample. | 1. Completeness of follow-up described. 2. Completeness of follow-up adequate. | 1. Appropriate follow-up to determine mortality. 2. Limited number of patients lost to follow-up. |
| 1. Prognostic factor of interest is adequately measured in study participants. | 1. Prognostic factors defined. 2. Prognostic factors appropriately measured. | i) SIRS/qSOFA scores calculated according to the standard definitions.  II) Measurement made on admission and recorded prospectively.  III) Missing values minimized and appropriately addressed. |
| 1. The outcomes of interest are adequately measured in study participants. | 1. Outcome defined. 2. Outcome measured appropriately. | i) Mortality.  II) 30-day mortality, in-hospital mortality or alternative outcome determined appropriately. |
| 1. Important confounders are accounted for. | 1. Confounders defined and measured. 2. Confounders accounted for. | Not applicable as prognostic scores are used independently. |
| 1. Appropriate statistical analysis. | 1. Analysis described. 2. Analysis appropriate. 3. Analysis provides sufficient presentation of data. | i) Uses and reports sensitivity/specificity /PPV and NPV or receiver operator characteristic curve for infection severity scores.  II) There is no selective reporting of results. |

Additional file 1- Hayden’s criteria for quality assessment, modified to apply to studies of infection.
